# Polyethylene Oxide as a Multifunctional Binder for High-Performance Ternary Layered Cathodes

**DOI:** 10.3390/polym13223992

**Published:** 2021-11-19

**Authors:** Jinshan Mo, Dongmei Zhang, Mingzhe Sun, Lehao Liu, Weihao Hu, Bing Jiang, Lihua Chu, Meicheng Li

**Affiliations:** School of New Energy, North China Electric Power University, Beijing 102206, China; mojinshan@ncepu.edu.cn (J.M.); zhangdongmei@ncepu.edu.cn (D.Z.); sunmingzhe@ncepu.edu.cn (M.S.); huweihao@ncepu.edu.cn (W.H.); jiangbing@ncepu.edu.cn (B.J.); chulihua@ncepu.edu.cn (L.C.)

**Keywords:** binder, ternary cathode material, lithium-ion battery, electrochemical performance

## Abstract

Nickel cobalt manganese ternary cathode materials are some of the most promising cathode materials in lithium-ion batteries, due to their high specific capacity, low cost, etc. However, they do have a few disadvantages, such as an unstable cycle performance and a poor rate performance. In this work, polyethylene oxide (PEO) with high ionic conductance and flexibility was utilized as a multifunctional binder to improve the electrochemical performance of LiNi_0.6_Co_0.2_Mn_0.2_O_2_ cathode materials. Scanning electron microscopy showed that the addition of PEO can greatly improve the adhesion of the electrode components and simultaneously enhance the integrity of the electrode. Thus, the PEO-based electrode (20 wt% PEO in PEO/PVDF) shows a high electronic conductivity of 19.8 S/cm, which is around 15,000 times that of the pristine PVDF-based electrode. Moreover, the PEO-based electrode exhibits better cycling stability and rate performance, i.e., the capacity increases from 131.1 mAh/g to 147.3 mAh/g at 2 C with 20 wt% PEO addition. Electrochemical impedance measurements further indicate that the addition of the PEO binder can reduce the electrode resistance and protect the LiNi_0.6_Co_0.2_Mn_0.2_O_2_ cathode materials from the liquid electrolyte attack. This work offers a simple yet effective method to improve the cycling performance of the ternary cathode materials by adding an appropriate amount of PEO as a binder in the electrode fabrication process.

## 1. Introduction

The utilization of nickel cobalt manganese ternary layered cathode materials can greatly increase the energy density and reduce the cost of lithium-ion batteries; however, they have several drawbacks, such as a poor rate performance, a quick capacity fading and a low initial Coulombic efficiency [1,2,3]. Body doping [4,5,6] and surface coating [7,8] are often used to modify the ternary cathode materials [9]. Yuan et al. [10] applied LiPF_6_ as a dopant to improve the cycling performance of LiNi_0.8_Co_0.1_Mn_0.1_O_2_ (NCM811) material, and found that the capacity and capacity retention rate of the 1 wt% LiPF_6_-doped cathode reached 162.3 mAh/g and 94.4% after 100 cycles at 0.5 C. Li et al. [11] synthesized FeF_3_-coated Li_1.2_Mn_0.54_Ni_0.13_Co_0.13_O_2_ cathode materials via a wet chemical process followed by a solid-state reaction, and the Coulombic efficiency, rate capability and cycling stability of the FeF_3_-coating materials were also greatly improved (e.g., 95% capacity retention after 100 cycles at 0.5 C). It is worth mentioning that both doping and coating can effectively improve the cycling performance of the ternary cathode materials, but they usually possess a few shortcomings, including complex operation procedures, such as blending and sintering, a high energy consumption and environmental pollution. Thus, there is always an urgent need to exploit simple, effective and environmentally friendly modification methods.

The electrodes in lithium-ion batteries are generally composed of active material, a polymer binder and a conductive agent, and the battery performance depends on the electrode components and contents. As a critical part of lithium-ion batteries, the binder only accounts for a small part of the electrode; however, it is responsible for tightly connecting the active material and the conductive agent with the current collector and for stabilizing the electrode structure, and thus it has an important impact on the battery performance [12]. The ideal binders for lithium-ion batteries should have a few basic characteristics: (1) a high binding capacity to bond the active material and the conductive agent; (2) a high corrosion resistance to the liquid electrolyte; (3) good flexibility and viscosity to buffer electrode volume change during the charge and discharge process; (4) other merits such as environmental friendliness and low cost. Polyvinylidene fluoride (PVDF) is a commonly used binder due to its high chemical, electrochemical and thermal stabilities. However, it has several problems, such as a low ionic conductivity and a poor adhesion ability, both of which seriously affect the electrochemical properties of the electrode materials [13,14,15]. Compared with the common modification methods of coating and doping, adjusting the type and content of the binder is a more convenient and environmentally friendly method, and can also effectively improve the battery cycling performance. A few researchers have been exploring novel binders to replace the traditional PVDF. Lei et al. [16] synthesized a water-based binder of lithium carboxymethyl cellulose (CMC-Li) using cotton as a raw material, and found that the capacity retention of the CMC-Li binder-containing LiFePO_4_ batteries reached 97.8% after 200 cycles. The rate performance was also greatly enhanced due to efficient lithium transport, a property of the CMC-Li binder. Eliseeva et al. [17] prepared LiFePO_4_-based cathodes using PEDOT: PSS/CMC as a composite binder, and the cathode exhibited a higher specific capacity and rate capability than the pristine cathode, owing to the combination of the high electronic conductivity of PEDOT:PSS and the ionic conductivity of CMC. 

**Figure 1:**
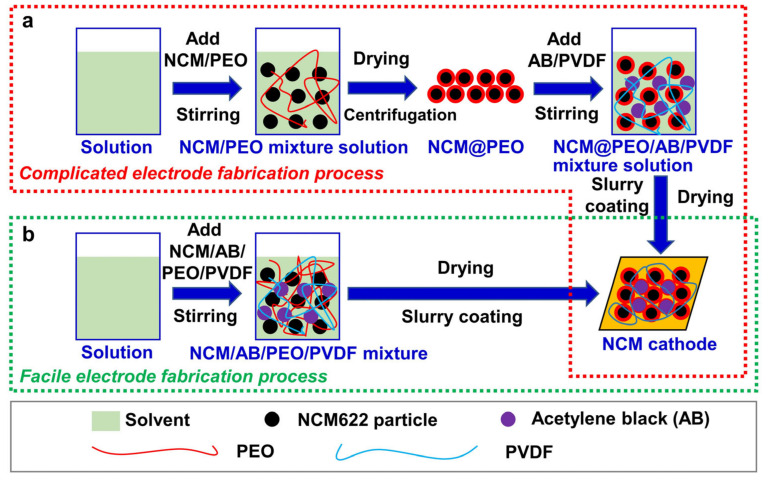
Schematic illustrations of the fabrication of NCM622 cathodes by (**a**) PEO coating on NCM622 particles and (**b**) by using PEO as a binder additive followed by slurry coating and drying processes.

Hence, the utilization of high-conductivity binders can significantly improve the battery cycling performance. The strategy also has other significant advantages, such as a simple operation process, environmental friendliness, and simple scale-up process. Polyethylene oxide (PEO) has the advantages of a good adhesive quality, high ionic conductivity, high flexibility, low cost, excellent processing property and environmental friendliness. Furthermore, Qiu et al. [18] found that PEO can be well-coated on the surface of silicon-based anode particles, which effectively reduces the electrochemical impedance of the batteries and prevents the breakage of the silicon anode particles. Therefore, PEO may be very suitable as a multifunctional binder for the ternary cathode materials. Herein, high ion conductivity and flexible PEO was applied as a multifunctional binder to partially replace the commonly used PVDF binder, to enhance the electrochemical cycling performance of LiNi_0.6_Co_0.2_Mn_0.2_O_2_ (NCM622) cathode materials (Figure 1b). Compared to the coating of PEO on the NCM622 particles (Figure 1a), this method of using PEO as an additive is more convenient and environmentally friendly for preparing the NCM622 cathodes (Figure 1b). Various characterizations further verified that the addition of 20 wt% PEO (of the PEO/PVDF binder) can greatly increase the conductivity of the cathode, improve the cathode integrity, reduce the battery impedance and protect the cathode materials from the liquid electrolyte corrosion, and thus enhances both the cycling stability and rate performance of the NCM622 cathode.

## 2. Materials and Methods

### 2.1. Preparation of PEO-Containing Electrodes

An amount of 0, 50, 80 and 100 mg of PVDF (Aladdin, Shanghai, China, the molecular weight is 1,000,000) were added to 4 bottles containing 4 mL N-methylpyrrolidone (NMP) (Aladdin, Shanghai, China) solutions and stirred for 10 min. Then, 100, 50, 20 and 0 mg PEO (Sigma Aldrich, Saint Louis, MO, USA, the molecular weight is 600,000) were added to the four solutions, respectively, and stirred at 45 °C for 6 h. A total of 10 mg graphene oxide (GO) (Tangshan Jianhua Science and Technology Development Co., Ltd. Tangshan, China) was also added to each solution, followed by ultrasonic dispersion for 2 h. Then, 90 mg acetylene black (AB) (Jinghong New Energy Company, Zhengzhou, China) was added, and the ultrasonic dispersion continued for another 2 h. A total of 800 mg of LiNi_0.6_Co_0.2_Mn_0.2_O_2_ (Tianjin Ivixin Chemical Technology Co., Ltd., Tianjin, China) was then added and stirred at 45 °C for 24 h to obtain four mixture solutions with different contents of PEO binder. The composite electrodes based on the PEO/PVDF binders were obtained by a 500 μm scraper coating method. The electrodes were dried at room temperature for 24 h, and then dried at 60 °C under a vacuum for another 24 h. The electrodes with 0, 20, 50 and 100 mg PEO (total PVDF/PEO content of 100 mg) were labeled as PEO0, PEO20, PEO50 and PEO100, respectively.

### 2.2. Preparation of PEO-Coated NCM622 Particle and PEO-Based Electrodes

Amounts of 0 and 0.917 g of PEO were added to 2 bottles containing 50 mL H_2_O, and then stirred at 45 °C for 12 h. A total of 500 mg of NCM622 was added to the two solutions, followed by ultrasonic dispersion for 2 h, and stirred at 45 °C for 12 h. The solutions were then centrifuged at 13,000 r/min for 10 min, and the lower layers of the two solutions were freeze-dried for 48 h to obtain the water-treated NCM622 and the NCM622@PEO particles.

An amount of 37.5 mg of PVDF was added to 2 bottles containing 1 mL N-methylpyrrolidone (NMP) solutions respectively and stirred for 30 min. Then, 7.5 mg graphene oxide (GO) was added to the two solutions, followed by ultrasonic dispersion for 2 h and then stirred for 8 h. After that, 30 mg acetylene black (AB) was added to both solutions and stirred for 12 h. An amount of 300 mg of water-treated NCM622 and PEO-coated NCM622 were added to the solutions and stirred for 24 h to obtain two mixture solutions. The composite electrodes were obtained by a 500 μm scraper coating method, before the electrodes were dried at room temperature for 24 h, and then dried at 60 °C under a vacuum for another 24 h. The NCM622 was 80 wt% of the composite electrode with a mass loading of around 7.0 mg/cm^2^.

### 2.3. Assembly of Lithium-Ion Batteries

The positive electrodes were cut into a small circle with a diameter of 12 mm by a tablet press machine, before the coin-type lithium-ion batteries were made in an argon-filled glove box, using the composite electrodes mentioned above and lithium foils as cathodes and anodes, respectively. The cathodes and the anodes were separated by a porous polyethylene/polypropylene diaphragm, and 1 M LiPF_6_ EC/DMC (200 μL was used in each cell) was used as the electrolyte.

### 2.4. Material Characterizations

A SU8010 field emission scanning electron microscope (SEM) (Hitachi, Tokyo, Japan) and a FEI F20 field emission transmission electron microscopy (TEM) (FEI Company, Hillsboro, OR, USA) coupled with an energy dispersive spectroscope (EDS) (FEI Company, Hillsboro, OR, USA) were used to observe the surface morphology and to analyze the elemental composition of the NCM622 particles and the different components of the composite electrodes. A D8 Focus X-ray diffractometer (XRD) (Karlsruhe, Germany) from Bruker AXS was used to explore the effect of PEO on the lattice structure of the ternary cathode material. To measure the ionic conductivity of the solid polymer electrolyte (SPE) film, the electrochemical impedance spectra (EIS) of the stainless steel (SS)/SPE/SS cells were first measured in a Zahner Zennium electrochemical workstation within 10^6^–10^−2^ Hz at an amplitude of 10 mV. The ion conductivity (*σ*) was calculated using an equation: *σ* = *L*/*SR*, where *R*, *S* and *L* were the resistance, surficial area and thickness of the SPE membranes, respectively [19]. The electronic conductivity of the electrodes was measured by an RTS-8 four-probe tester from Guangzhou Instrument Company. A Zahner Zennium electrochemical workstation was also utilized to measure the impedance of the NCM622/Li batteries. Land CT2001A battery test systems were applied to measure the cycling performance of the lithium-ion batteries at various charge-discharge rates (1 C = 180 mA/g). The software of ZsimDemo were used to calculate the electrochemical resistance values based on the equivalent circuit model.

## 3. Results and Discussion

### 3.1. Preparation and Properties of the PEO-Based Electrolytes and NCM622 Particles

**Figure 2:**
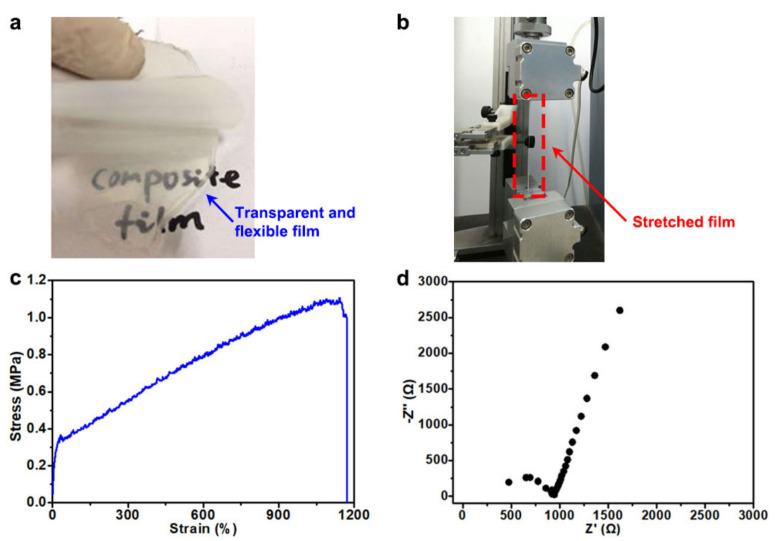
Optical images of a PEO-LiTFSI solid electrolyte film (**a**) without and (**b**) after the tensile measurement; (**c**) tensile stress–strain curve and (**d**) electrochemical impedance spectra at room temperature of the PEO-LiTFSI electrolyte film.

PEO-LiTFSI solid electrolyte films were prepared by a solution casting method (Figure 2a) to prove the ionic conductance of PEO and its efficacy in coating the NCM622 particles (the experimental detail can be seen in [19]). The PEO-LiTFSI electrolyte films had high transparency and flexibility, and cannot break up, even after repeated stretch and bending processes (Figure 2b). The mechanical tensile strength of the PEO-LiTFSI electrolyte film can reach 1.1 MPa (Figure 2c) with a high tensile strain of nearly 1200%, which is expected to greatly alleviate the volume change in the ternary electrode particles in the process of charge and discharge, and meanwhile stabilize the morphology of the positive electrode. Further, it was found that the electrolyte membrane with a thickness of 286 µm (LiTFSI content of ~25 wt%) showed a high ionic conductivity of 1.6 × 10^−5^ S/cm (resistance of ~922 Ω) at room temperature (Figure 2d). It should be noted that when PEO was used as a binder to prepare the electrodes, carbon black nanoparticles, NCM622 particles and other components would function as fillers to reduce the crystallinity of PEO and further increase the ionic conductivity [19].

**Figure 3:**
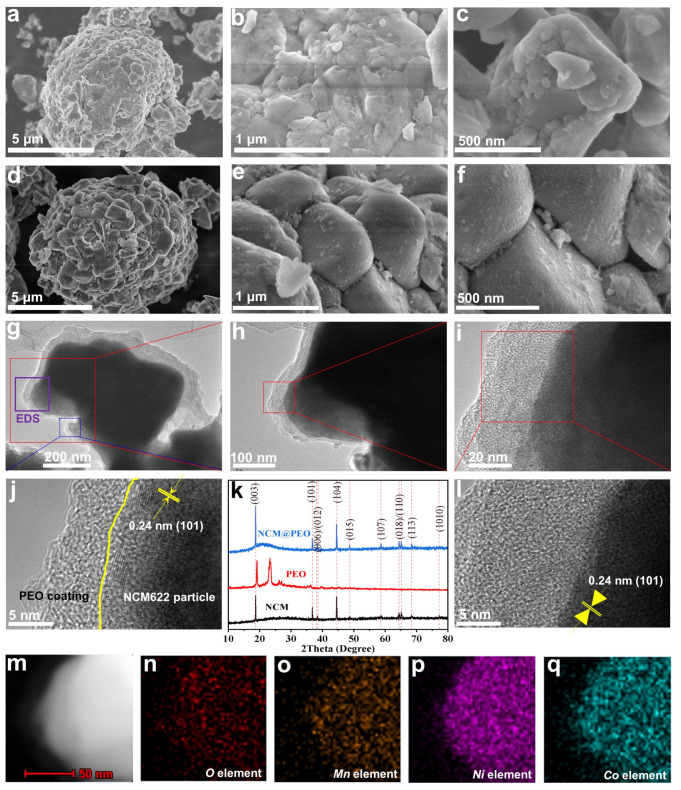
(**a**–**c**) SEM image of the pristine NCM622 particles and (**d**–**f**) the PEO-coated NCM622 particles, (**g**–**j**,**l**) TEM images of the PEO-coated NCM622 particles, (**k**) XRD patterns of the pristine NCM622 particles, PEO and the PEO-coated NCM622 particles, and (**n**–**q**) the EDS mapping of the elements of O, Mn, Ni and Co in the corresponding TEM image (**m**), respectively.

Moreover, the NCM622 particles coated with PEO were prepared by solution stirring, centrifugation and drying processes. From the SEM images, we can see that the NCM622 particles almost maintained the secondary particle morphology (i.e., the large particles were composed of small particles of several hundred nanometers in diameter), but a few NCM622 particles were broken during the SEM sample preparation process (Figure 3a–c), which should be ascribed to the low mechanical strength between the primary nanoparticles. In addition, the surfaces of the primary particles were relatively smooth (Figure 3c). In stark contrast, PEO was evenly covered on the surface of the positive NCM622 particles, and the surface became much coarse (Figure 3d–f). All the NCM622 particles remained the secondary particle morphology, which may be attributed to the binding effect of the flexible PEO coating. TEM images furtherly proved that PEO was coated on the NCM particles, and the PEO coating layer showed an average thickness of 35 nm (Figure 3g). The high-resolution TEM image also disclosed a lattice space of around 0.24 nm, corresponding to the (101) crystal face (Figure 3j,l). The O, Mn, Ni and Co element mapping images (Figure 3n–q) further confirmed the PEO coating layer (mainly the O element) on the NCM622 particle (mainly the metal elements) surface. These results clearly indicate that PEO is an excellent multifunctional binder with good adhesive capability, a high ionic conductivity and high flexibility. XRD characterizations showed that PEO had obvious crystallization peaks of around 20° and 25°, indicating that the pristine PEO powder mainly existed in the form of crystallization (Figure 3k). Nevertheless, the PEO-coated NCM622 (NCM622@PEO) particles did not show strong diffraction peaks around 20° and 25°, indicating that PEO mainly existed in the form of an amorphous state, and that this would enhance the kinetic ability of the PEO chain segments significantly and help to increase the ionic conductivity [20]. There were obvious diffraction peaks at 18.6°, 36.7°, 38.3°, 44.5°, 48.6°, 58.6°, 66.4°, 65.0° and 68.3° in the pristine NCM622 particles, which corresponded to the crystal faces of (003), (101), (012), (104), (015), (107), (018), (110) and (113), respectively (Figure 3k). It can be also seen that the PEO-coated NCM622 (NCM622@PEO) particles had almost the same number and intensity of the diffraction peaks as the pristine NCM622 particles, indicating that the coating of PEO did not change the lattice structure of the ternary cathode materials. The amorphous layers of PEO coating on the surface of the cathode particles are also expected to improve the ionic conductivity of the entire electrode and prevent the corrosion of the liquid electrolyte on the ternary NCM622 cathode materials [18].

### 3.2. Micromorphology of the PEO-Containing Cathodes

**Figure 4:**
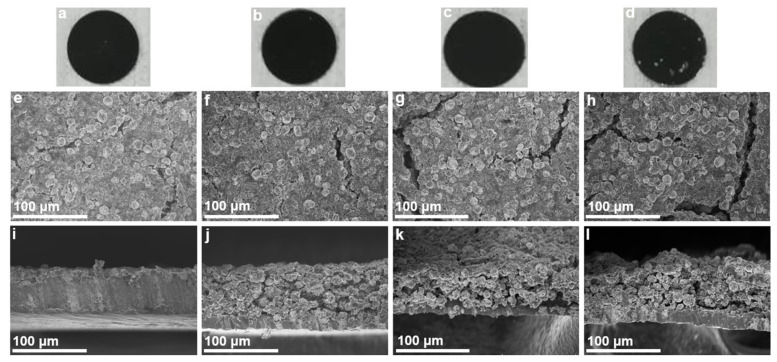
(**a**–**d**) Optical images, and (**e**–**h**) surficial and (**i**–**l**) cross-sectional SEM images of the (**a**,**e**,**i**) PEO0, (**b**,**f**,**j**) PEO20, (**c**,**g**,**k**) PEO50, and (**d**,**h**,**l**) PEO100 cathodes.

The PEO binder-containing NCM622 cathodes (12 mm in diameter) were further prepared by the traditional scraper coating method, and the effect of the PEO content on the electrode morphology was investigated by the optical and electron microscopy characterizations. According to the optical images (Figure 4a–d), the PEO20 electrode had no obvious cracks and the electrode components were evenly distributed, while the PEO0 and PEO100 electrodes had several obvious cracks, indicating that the addition of the appropriate amount of PEO could significantly improve the adhesion between the electrode components and the structural consistency of the whole electrode. Further SEM characterizations (Figure 4e) showed that the PEO0 electrode had only a few obvious cracks on the surface; however, the cross-section photo showed that the electrode materials had fallen off from the aluminum foil (Figure 4i), which also verified the poor adhesive ability of the PVDF binder. In stark contrast, the PEO20 electrode had a small number of cracks on the surface (Figure 4f) and the electrode materials had good physical contact with the aluminum foil (Figure 4j). However, when the PVDF was completely replaced with PEO as the binder, more cracks appeared on the electrode surface (Figure 4h), and more pores were also generated at the cross-section (Figure 4l), which may be attributed to the high bond strength of PEO and the resulted agglomeration and uneven dispersion of the electrode particles. Nevertheless, compared with the PEO0 electrode (Figure 4i), the binding ability of the electrode materials to the aluminum foil was significantly enhanced. In short, an appropriate amount of PEO can increase the adhesive ability among the electrode particles and can also enhance the bonding strength between the electrode particles and the aluminum foil, improving the structural integrity and stability of the whole electrode.

### 3.3. Electrochemical Properties of the PEO-Containing Batteries

**Figure 5:**
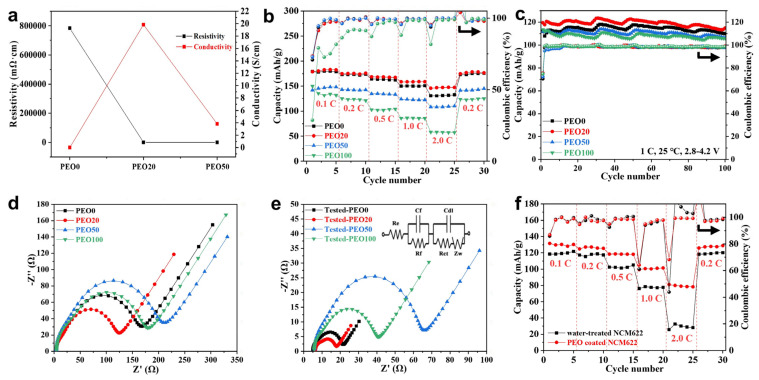
(**a**) Electronic resistivity and conductivity of the PEO0, PEO20 and PEO50 electrodes; (**b**) rate performance of the NCM622/Li batteries; (**c**) cycling performance of the NCM622/Li batteries at 1 C; electrochemical impedance spectra of the batteries (**d**) before and (**e**) after the 100 charge–discharge test (The inset in (**e**) is an equivalent circuit model for the half-cells); (**f**) rate performance of the NCM622/Li batteries using water-treated NCM622 particles and PEO-coated NCM622 particles, respectively.

The effect of the PEO content on the electronic conductivity of the NCM622 cathodes was studied by four-probe method. As can be seen from Figure 5a, the resistivity of the electrode without PEO was 783.0 Ω·cm, and the corresponding electronic conductivity was calculated as 0.00128 S/cm. When the PEO content was 20 wt% (of the PEO/PVDF binder), the resistivity of the electrode sharply decreased to 50.0 mΩ·cm, and the corresponding conductivity was calculated to be as high as 19.8 S/cm, indicating that increasing the PEO content would lead to a large increase in the electrode conductance. It can be seen from Figure 4 that the PEO20 electrode had a good overall structural consistency, fewer cracks and strong bonding with the aluminum foil, which means that the PEO20 electrode conductivity was much higher than that of the PEO-free electrode. However, excessive PEO would result in an increase in the cracks and pores in the electrode, and thus the PEO50 and PEO100 electrodes showed a much lower conductivity (note that the resistivity of the PEO100 electrode cannot be detected by the RTS-8 four-probe tester, Figure 5a).

The NCM622/Li coin-type batteries were tested at various charge-discharge rates in order to verify the effect of the PEO binder. As can be seen from Figure 5b, the discharge capacities of the PEO-free battery at 0.2 C, 0.5 C, 1 C and 2 C were lower than the PEO20-based battery. In particular, the PEO20 battery displayed a discharge capacity of 147.3 mAh/g, which was much higher than the PEO0 battery (131.1 mAh/g) at 2 C. However, the charge and discharge capacities of the PEO50 and PEO100 batteries were lower than those of the PEO20 battery, which can be ascribed to the uneven dispersion and agglomeration of the electrode materials caused by the high PEO content, and the resulting reduction of the structural consistency and conductivity of the electrodes (Figure 4 and Figure 5a). It can be also seen that the PEO20 battery showed a much higher capacity than other batteries when increasing the charge and discharge rate, further indicating the fast charge transfer kinetics behavior in the PEO20 cathode.

As can be seen from Figure 5c, the PEO20 electrode had the best cycling performance among the electrodes. The PEO20 electrode had an initial discharge capacity of 119.5 mAh/g and a Coulombic efficiency of 75.6% at 1 C, which were higher than those of the PEO0, PEO50 and PEO100 electrodes (111.9 mAh/g and 70.5%, 112.0 mAh/g and 72.4%, and 113.6 mAh/g and 74.8%, respectively). The PEO20 electrode also exhibited a discharge capacity of 114.8 mAh/g after 100 cycles at 1 C, which was also higher than the PEO0, PEO50, and PEO100 electrodes (109.9, 106.3 and 105.8 mAh/g, respectively). Moreover, the capacity retention of the PEO20 electrode was as high as 96.1%. The greatly enhanced cycling performance of the PEO20 electrode should have benefited from the better structural integrity (Figure 4b,f,j) higher electrical conductivity (Figure 5a) of the PEO20 electrode, and the excellent flexibility of the PEO electrolyte coating layers on the NCM622 particles (Figure 3f–j,l), which can effectively alleviate the volume change of the NCM622 particles during the charge–discharge processes and maintain the structural stability of the whole electrode [18,21].

Electrochemical impedance measurements were also conducted to disclose the effect of the PEO binder on the battery impedance (Figure 5d,e). The circles in high- and medium-frequency regions were usually attributed to the SEI formation and contact resistance, and charge-transfer impedance at the electrolyte/electrode interface, respectively [21,22]. An equivalent circuit model was also utilized for the ZsimDemo software simulation and calculation. *R_e_* and *Z_w_* are the electrolyte resistance and Warburg impedance, respectively. *R_f_* and *C_f_* are the resistance and capacitance regarding the SEI film, respectively. *R_ct_* and *C_dl_* are the charge-transfer resistance and double-layer capacitance, respectively. We can see that the total impedances of the PEO20 battery before and after when the long-term charge and discharge cycling (125.0 and 18.0 Ω) were much lower than the PEO-free (168.0 and 21.6 Ω) and other PEO-based PEO50 and PEO100 batteries. Especially, the *R_e_* and the *R_ct_* of the PEO-free battery before the long-term cycling were 4.6 and 25.0 Ω, respectively, but the *R_e_* and *R_ct_* of the battery decreased greatly with the addition of the PEO binder (*R_e_* and *R_ct_* of the PEO20 battery were 3.8 and 16.3 Ω, respectively). These data indicated that the addition of PEO can effectively protect the NCM622 particles from liquid electrolyte attack and reduce the overall battery impedance, and thus greatly improve the cycling performance of the batteries, which is consistent with the previous SEM and conductivity results. We can also see that the PEO100 battery showed lower total impedances than the PEO50 battery, which may be ascribed to the high porosity of the PEO100 electrode (Figure 4h,l). The reason why the electrochemical resistances decreased during the charge–discharge process also needs to be further investigated in future study. 

The water-treated NCM622 particles and the PEO-coated NCM622 particles were also used to prepare NCM622/Li coin-type cells for comparison with the batteries using PEO as a binder additive. As can be seen in Figure 5f, the battery using the NCM622 particles washed with water showed discharge capacities of 119.6, 117.4, 102.6, 77.4 and 29.1 mAh/g at 0.1 C, 0.2 C, 0.5 C, 1 C and 2 C, respectively. These specific capacities were much lower than the measurement results of the pristine NCM622 particle-based coin cells (Figure 5b), indicating that water treatment has an adverse effect on the cycling performance of the NCM622. In comparison, the coin cells that used the PEO-coated NCM622 particles showed much higher discharge capacities (130.0, 126.1, 118.4, 100.5, and 79.4 mAh/g at 0.1 C, 0.2 C, 0.5 C, 1 C and 2 C, respectively) than the water-treated NCM622 particle-based cells (Figure 5f). However, these rate capacities were still lower than those (181.4, 175.0, 168.0, 158.6, 147.0 mAh/g at 0.1 C, 0.2 C, 0.5 C, 1 C and 2 C, respectively) of the PEO20-based battery (Figure 5b), further proving that using PEO as a binder additive is a simple yet effective method to improve the cycling performance of the ternary cathode materials.

## 4. Conclusions

The NCM622 electrodes based on the PEO electrolyte were prepared by a simple and environmentally friendly method, and the effect of the PEO content on the microstructure and electrochemical performance of the electrodes was investigated. The PEO binder can not only be uniformly coated on the surface of the NCM622 particles to protect the electrode particles, but also can effectively improve the structural integrity and conductivity of the whole electrode and reduce the NCM622/Li battery impedance. When the PEO content was 20 wt% of the PEO/PVDF binder, the discharge capacity of the battery at 2 C increased by 12.4% compared with the PEO-free battery, and the PEO20 battery also showed better cycling stability. The discharge capacity of the PEO20 battery was 114.8 mAh/g after 100 charge and discharge cycles at 1 C, higher than that of the PEO-free battery, and the capacity retention of the PEO20 battery was also as high as 96.1%.

## Data Availability

The data presented in this study are available on request from the corresponding author.
